# Population structures of Brazilian tall coconut (*Cocos nucifera* L.) by microsatellite markers

**DOI:** 10.1590/S1415-47572010005000077

**Published:** 2010-12-01

**Authors:** Francisco E. Ribeiro, Luc Baudouin, Patricia Lebrun, Lázaro J. Chaves, Claudio Brondani, Maria I. Zucchi, Roland Vencovsky

**Affiliations:** 1 Embrapa Tabuleiros Costeiros, Aracaju, SEBrazil; 2 CIRAD, Amélioration Cocotier, MontpellierFrance; 3 Universidade Federal de Goiás, Escola de Agronomia, Goiânia, GOBrazil; 4 Embrapa Arroz e Feijão, Santo Antônio de Goiás, GOBrazil; 5 Instituto Agronômico de Campinas, Campinas, SPBrazil; 6 Departamento de Genética, Escola Superior de Agricultura Luiz de Queiróz, Universidade de São Paulo, Piracicaba, SPBrazil

**Keywords:** coconut, genetic diversity, genetic variability, molecular markers, SSR

## Abstract

Coconut palms of the Tall group were introduced to Brazil from the Cape Verde Islands in 1553. The present study sought to evaluate the genetic diversity among and within Brazilian Tall coconut populations. Samples were collected of 195 trees from 10 populations. Genetic diversity was accessed by investigating 13 simple sequence repeats (SSR) loci. This provided a total of 68 alleles, ranging from 2 to 13 alleles per locus, with an average of 5.23. The mean values of gene diversity (H_e_ ) and observed heterozygosity (H_o_ ) were 0.459 and 0.443, respectively. The genetic differentiation among populations was estimated at ${\hat{\theta }}_{P}=0.1600$and the estimated apparent outcrossing rate was t_a_ = 0.92. Estimates of genetic distances between the populations varied from 0.034 to 0.390. Genetic distance and the corresponding clustering analysis indicate the formation of two groups. The first consists of the Baía Formosa, Georgino Avelino, and São José do Mipibu populations and the second consists of the Japoatã, Pacatuba, and Praia do Forte populations. The correlation matrix between genetic and geographic distances was positive and significant at a 1% probability. Taken together, our results suggest a spatial structuring of the genetic variability among the populations. Geographically closer populations exhibited greater similarities.

## Introduction

Two main groups of coconut palm trees (*Cocos nucifera* L.), the Tall (*Typica*) and the Dwarf (*Nana*) types, are known. Coconut is the most widely naturally distributed palm tree. It is extensively cultivated around the world and is considered to be one of the most important tropical species used by man (Persley, 1992). Southeastern Asia is believed to be the center of origin of the species due to the great morphological variability, the large number of popular/local names and plant uses, and the number of associated insects in that region (Persley, 1992). It has been suggested that the spreading of the species throughout diverse regions of the world occurred naturally, carried by oceanic currents from Southeast Asia to the Pacific and Indian oceans and by human migration during the colonization of Asia and America (Harries, 1978). The introduction of the species from the Atlantic coast of Africa to America occurred after the discovery of the Cape of Good Hope (Purseglove, 1975), during the period of extensive mercantile navigation in the 16^th^ century.

The Tall group was introduced to Brazil from the Cape Verde Islands in 1553. Plants of this group exhibit a later reproductive stage than those of the Dwarf group. The reproductive cycle begins after approximately five to seven years, producing a substantial number of large fruits, primarily from cross-fertilization (Siqueira *et al.*, 1998).

In Brazil, the vast majority of coconut palms are located in the Northeast, where populations of Tall coconut that are more than 80 years old are found. These populations may represent an excellent source of adapted germplasm for breeding programs. However, little is known about their genetic variability.

Tall coconut palm trees have been growing in Brazil for more than 450 years. Nowadays, the species is distributed along the coast, from the equator to the Tropic of Capricorn (approximately 23°26'17” south of the equator), with the majority of the plants located on the Northeastern coast. These populations are considered to have adapted to distinct environmental conditions and have undergone genetic divergence (Ribeiro *et al.*, 1999), forming ecotypes of the Tall group.

In contrast to isoenzyme (Benoit H and Ghesquière M, Rapport interne IRHO-CIRAD, FAR), and leaf polyphenol investigations (Jay *et al.*, 1989), which have led to inconclusive results, genetic markers based on DNA are considered to be the most acceptable tool for the study of genetic diversity in the coconut (Lebrun *et al.*, 1995). Further studies have provided a better understanding of the genetic diversity in several Tall and Dwarf coconut populations by employing Random Amplification of Polymorphic DNA (RAPD) markers (Wadt *et al.*, 1999). More recently, the quantitative trait loci (QTL) involved in wax component production were mapped in a controlled-cross population of Tall genotypes, using amplified fragment polymorphism (AFLP) and simple sequence repeats (SSR) markers (Riedel *et al.*, 2009). Similarly, SSR markers have also been shown to be a powerful tool in studies of population structure, due mainly to their multiallelic and highly polymorphic sequences and their ability to be amplified by polymerase chain reaction (PCR) (Chase *et al.*, 1996; Morgante *et al.*, 1996).

In this study we investigated the genetic diversity of 10 populations of Brazilian Tall coconut trees, employing 13 SSR loci to characterize their genetic variability, population structure, and reproductive system.

## Material and Methods

### Plant material

For the current study, typical populations of the Tall group of coconut palm trees were chosen based on legitimacy, homogeneity, and isolation criteria. Legitimacy was based on the population age. Because the Dwarf group was introduced in Brazil in 1925, only individuals older than 80 years were selected as representatives of the Tall group, thus preventing the inclusion of natural hybrids between the groups. According to the homogeneity criterion, populations exclusively composed of trees from the Tall group were selected. Finally, according to the isolation criterion, we sampled only populations that are 1,000 m distant from Dwarf palm groups or that are 500 m distant plus an intervening stretch of vegetation.

The populations described in Table 1 were identified in Brazil as genuine and homogeneous representatives of the Tall group and in adequate conditions of isolation.

### DNA extraction

Leaflet segments of approximately 50 cm in length were taken from the youngest leaf of each sampled tree. The DNA was extracted according to the modified CTAB protocol adapted for coconut (Lebrun *et al.*, 1998; Baudouin and Lebrun, 2002). The DNA concentration was determined by automatic fluorimetric quantification (number of evaluated trees per population, see Table 1).

### SSR analysis

For the PCR reaction, a final volume of 25 μL was prepared. It contained a mixture of 2.5 μL of 10X PCR buffer, 2.0 μL of dNTP (2 mM of each dNTP), 0.25 μL of MgCl_2_ (50 mM stock), 0.5 μL of forward primer (10 μM stock), 0.5 μL of reverse primer (10 μM stock), 0.5 μL of Taq DNA Polymerase (2 U/μL), 5 μL of genomic DNA (2.5 ng/μL), and 13.75 μL of sterile water. PCR reaction cycles consisted of an initial denaturation step at 94 °C for 5 min, followed by 36 cycles at 94 °C for 30 s for denaturation, one minute at 51 °C for primer annealing, and one minute at 72 °C for extension, plus an additional final extension step of 5 min at 72 °C.

Thirteen fluorescence-labeled primer pairs that were designed and selected by Baudouin and Lebrun (2002) were used for SSR amplification. The amplified fragments were resolved on polyacrylamide gels employing a LICOR IR2 4200 sequencer. The gels were scored and the individuals were genotyped according to allele size (number of base pairs) in comparison to a standard marker (1 kb).

### Statistical analysis

The structuring of the genetic variability was evaluated employing *F*, θ_p_, and *f* parameters (Weir and Cockerham, 1984), which are analogous to the Wright (1951) *F*_*IT*_, *F*_*ST*_, and *F*_*IS*_ statistics, respectively. Estimates of the parameters were obtained using the Genetix 4.03 software (Belkhir *et al.*, 2001). The parameter *R*_*ST*_ (Slatkin, 1995), which is an analogue to θ_p_ and *F*_*ST*_, was also calculated in order to obtain the interpopulation genetic differentiation rate for comparison purposes. Originally, the parameter was estimated considering a stepwise mutation model, a condition not assumed for θ_p_ or *F*_*ST*_, thus tending to underestimate differentiation as the model prevails (Hardy *et al.*, 2003). The *R*_*ST*_Calc software (Goodman, 1997) was used to calculate *R*_*ST*_ estimates. Confidence intervals at 95% probability were obtained for the parameters by bootstrapping 10,000 replicates. The observed heterozigosity (H_o_) and gene diversity H_e_ (Nei, 1973) were calculated for each individual population.

To estimate *F*, θ, and *f*, a random model was assumed so that the sampled populations are considered to be local representatives of the species and thus are assumed to have a common evolutionary history (Weir, 1996).

For each investigated locus, we did the adherence test to the Hardy-Weinberg proportions, according to Weir (1996), and using the TFPGA software (Miller, 1997) by the conventional Monte Carlo method with 10 batches and 1,000 permutations per batch. The apparent outcrossing rate (t_a_) was obtained by the fixation index *f* for each population, assuming mating system equilibrium (Vencovsky, 1994), so that t_a_ = (1-*f*)/(1+*f*).

Nei (1972) genetic distances were estimated for population pairs and used to the neighbor-joining cluster analysis (Saitou and Nei, 1987), employing the PHYLIP 3.6 software (Felsenstein, 2004). In order to visually represent the pattern of divergence among the populations, an unrooted dendrogram was constructed. Genetic distances were also correlated to the corresponding geographic distances and the significance of Pearsons correlation coefficient was tested according to the Mantel procedure.

In addition, the software STRUCTURE (Pritchard *et al.*, 2000) was used to investigate the population structure, using a burn-in of 10,000, a run length of 100,000, and a model that allowed admixture and correlated allele frequencies. Ten independent runs yielded consistent results.

## Results

The total number of investigated alleles, gene diversity (H_e_), and observed heterozygosity (H_o_) for each SSR locus are shown in Table 2. The combination of 13 SSR loci generated a total of 68 alleles, with a mean of 5.23 alleles per locus and ranging from two (CnCir E12) to 13 alleles (CnCir E2). The loci CnCir A3 and CnCir E2 presented the lowest (0.036) value and the highest value (0.671), respectively, for observed heterozygosity, with a mean value of 0.443 for the 13 investigated loci in the studied populations. Monomorphic loci were absent from the studied sample. Gene diversity ranged from 0.034 for the locus CnCir A3 to 0.711 for the locus CnCir E2, with an overall mean of 0.459.

Estimates of parameters that were related to the genetic structure of the populations were: 
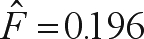
, 
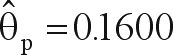
, and 
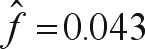
 (Table 3). Considering that the confidence intervals did not include zero, the hypothesis that the respective parameters differ from zero was accepted.

The correlation between the matrix of genetic distances and the geographic distances among the 10 populations studied was r = 0.598, which is statistically significant (p = 0.0027), according to the Mantel test. The smallest distances were found between the populations of Bahia Formosa and Georgino Avelino (0.034) and Georgino Avelino and São José do Mipibu (0.035). Relatively small distances were also found for the populations of Japoatã and Pacatuba (0.063), Baia Formosa and São José do Mipibu (0.071), Pacatuba and Praia do Forte (0.096), and Japoatã and Praia do Forte (0.099). The greatest genetic distances were observed between the populations of Santo Inácio and Georino Avelino (0.390), Santo Inácio and São José do Mipibu (0.381), Merepe and Praia do Forte (0.343), Merepe and Pacatuba (0.335), and Luis Correia and Pacatuba (0.335). The distance comparison results are summarized in Table 4.

Estimates of the fixation index (*f*) for each population and the corresponding confidence intervals are given in Table 5. These values ranged from -0.100, for the population Pacatuba (9), to 0.134 for the population São José do Mipibu (5), with an overall mean of 0.043. The estimates did not significantly differ from zero, except for the population of Pacatuba ( 
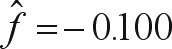
), which displayed a high frequency of heterozygous individuals. The corresponding values of apparent outcrossing rates (t_a_) are also shown in Table 5. They ranged around the overall mean of 0.918, which was statistically significant at a 5% probability in comparison to 1.0.

Of the 130 tests for Hardy-Weinberg equilibrium (13 loci in 10 populations), only 16 were statistically significant. For most of the studied populations, statistical significance was found for only one or two loci. The exception was the population of Baia Formosa with four loci that exhibited a significant departure from the Hardy-Weinberg proportions among the 13 loci.

**Figure 1:**
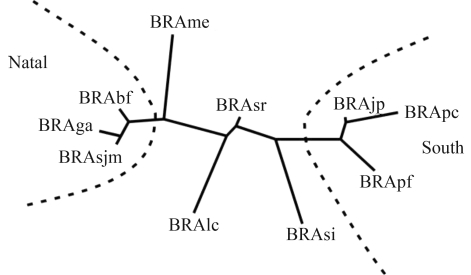
Genetic divergence pattern among ten populations of Brazilian Tall coconut, obtained by the neighbor-joining method based on genetic distances (Nei, 1972).

The pattern of genetic divergence among the investigated populations of Brazilian Tall coconut that were obtained from the Nei genetic distance is shown as a dendrogram in Figure 1. Data analysis showed a divergent pattern among 10 populations, revealing that the populations of Baía Formosa, Georgino Avelino, and Sao Jose do Mipibu are genetically similar and represent a distinct group in comparison to the other populations. These populations are located in the proximity of the city of Natal (RN). A similar situation is observed for Japoatã, Pacatuba, and Praia do Forte populations that also exhibited genetic similarities among themselves, clustering as a second group, which was denominated the Southern group due to its location. The remaining populations exhibit varying divergence patterns.

**Figure 2:**
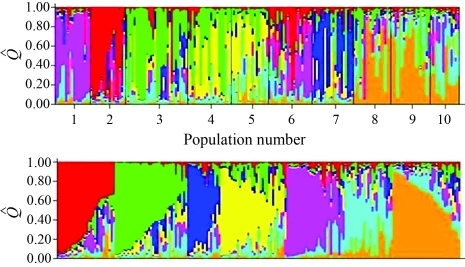
Population structure analysis based on multilocus genotyping data of the ten investigated Tall coconut populations. The first panel refers to a K established for seven groups. The second panel refers to K established for seven groups separated by $\hat{Q}$ ( $\hat{Q}$: estimated membership coefficient for each individual).

Clustering of individuals was done using the Structure software at *K* = 7 (Figure 2). Individuals are represented by vertical colored lines. The same color in distinct individuals indicates that they are from the same cluster. Different colors in the same individual indicate the percentage of the genome that is inherited from each cluster. Structure analysis (Figure 2) and the dendrogram (Figure 1) were congruent, as clustering gave rise to the same groups, namely: the Natal group, consisting of the populations of Baia Formosa, Georgino Avelino, and São José do Mipibu; and the Southern group, consisting of the populations of Japoatã, Pacatuba, and Praia do Forte (Figure 2).

## Discussion

Of the 68 alleles detected, four could be considered to be localized and common, since they were found in a unique population, although with a frequency = 5% (Perera *et al.*, 2001). To include these alleles in our analysis, the strategy of collecting relatively small samples in a large number of populations could be employed. Breeding efforts are often concentrated on this category of allele, since disperse common alleles are present even in small samples collected from a few populations (Marshall and Brown, 1975).

In the present study, the mean number of alleles per locus (5.2) was similar to that found in other studies of coconut palm tree populations using SSR markers. Rivera *et al.* (1999), using 38 SSR loci, found an average of 5.2 alleles per locus and a range of 2 to 9 alleles in a total of 198 SSR markers. Perera *et al.* (2000), using 8 SSR loci, found an average of 6.3 alleles and a range of 3 to 9 in a total of 50 alleles. Konan *et al.* (2007) evaluated gene diversity in 21 genotypes of three coconut accessions and detected a total of 68 alleles at 13 microsatellite loci. The number of alleles ranged from 3 to 7, with an average of 4.83 alleles. Gene diversity ranged from 0.475 to 0.832, with an average of 0.686. The extent of genetic diversity in 26 coconut accessions from the Andaman and Nicobar Island was determined using 14 microsatellite markers. A total of 103 alleles were detected with an average of 7.35 alleles per locus, and average observed and expected heterozigosity of 0.29 and 0.66, respectively (Rajesh *et al.*, 2008).

The gene diversity in the present study (H_e_ = 0.459) was lower than that found by Perera *et al.* (2001) in *ex situ* collections of Tall coconut trees in Sri Lanka, with values ranging from 0.426 to 0.846 and an average of 0.682. The maximum possible value of gene diversity (H_e_ max) within a population, for a locus with A alleles, is H_e_(max) = (A-1)/A. With A between 5 and 6, this value is 0.80 and 0.83, respectively, thus indicating that the investigated populations exhibited approximately 56% of the maximum, as a consequence of uneven values of allelic frequencies per locus within the populations.

The estimated intrapopulation fixation index (*f*), despite being significantly different from zero, was small in magnitude (0.043), indicating a predominantly panmixia reproduction system among the populations. The low inbreeding rate detected may have resulted from intermating of related parents or from natural self-fertilization. Further insights on the predominant reproductive system of the investigated populations would require data from the offspring of maternal families. Considering the overall apparent outcrossing rate t_a_ = 0.92 and, in the case of inbreeding by selfing, the apparent rate of self-fertilization is very small ( 
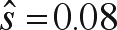
 or 8%).

The value of total fixation was relatively high ( 
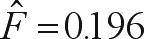
) and caused primarily by the considerable degree of genetic divergence among populations ( 
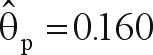
). The estimate of R_ST_ (0.086) was approximately one half of the corresponding θ_P_ value, although with a slightly overlapping confidence interval. These observations may indicate that the stepwise model is not the most appropriate means to explain the recent evolution of the populations. In fact, the allelic frequencies suggest an independent size distribution for the investigated loci. Therefore, estimate θ_P_ was considered to be more adequate to represent differentiation among populations in the present study. The Nei (1972) interpopulation parameter was also estimated for comparison purposes and 
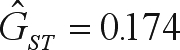
 was found to be similar to estimated θ_P_.

The genetic divergence was evaluated from a cluster analysis based on genetic distances, where the populations were grouped on the basis of similarity. The populations of Baia Formosa, Georgino Avelino, and São Jose do Mipibu were clustered as group 1 and labeled Natal; whereas the populations of Japoatã, Pacatuba, and Praia do Forte were clustered in group 2 and denominated Southern. The remaining populations showed erratic patterns of divergence (Figure 1).

The results of the structure analysis (Figure 2) were consistent with the dendrogram (Figure 1). The populations clustered similarly, forming two major groups, namely group 1 (Natal group) and group 2 (Southern group) (Figure 2). Both analyses of the genetic structure of the studied coconut populations, distance and Bayesian, demonstrated that the interpopulational genetic divergence is spatially structured and probably in a clinal variation pattern. These results are corroborated by the observed correlation between the genetic matrix and geographic distances (r = 0.598), which is considered to be intermediate to high.

Considering the model where a large population is split into subpopulations, the effective sampling size of individual or seed samples is inversely proportional to θ_P_ (or F_ST_) (Vencovsky and Crossa, 1999). For high values of this parameter, as observed here, a large number of subpopulations must be sampled in order to reach an adequate effective size for *ex situ* or *in situ* conservation programs.

These results, along with historical records, suggest that the populations are undergoing a recent process of differentiation, meaning that a few generations have passed in the process of evolution from the ancestor populations. The first record of the introduction of coconut palms in Brazil dates back to 1553, as mentioned previously. However, individuals from the species can live from 80 to 100 years. This indicates that there have been relatively few generations in Brazil and that the present structuring is probably due to genetic drift, strongly influenced by a founder effect, and possibly due to indirect effect of artificial selection by humans.

The correlation between the genetic and geographic distances matrix (r = 0.598; p = 0.0027) may be considered to be intermediate to high, demonstrating that interpopulational genetic divergence is structured spatially and probably in a clinal variation pattern. These analyses indicate that a stochastic process is probably responsible for the differentiation, with genetic drift only partially counter-balanced by short-distance gene flow.

The data suggest the possibility of clustering of the populations with genetic distances inferior to 0.1. Two population groups were formed: group 1 composed by the populations of Baia Formosa, Georgino Avelino, and São José do Mipibu; and group 2, consisting of the populations of Japoatã, Pacatuba, and Praia do Forte. Our data evidenced that the genetically most similar populations were also geographically closest. The differentiated pattern of the other populations did not allow consistent clustering (Figure 1).

The results of the structure analysis were consistent with the dendrogram. The groups obtained resulted from similar clustering, namely: group 1, consisting of the population of Baia Formosa, Georgino, Avelino and São José do Mipibu and denominated Natal group; and group 2, consisting of the populations of Japoatã, Pacatuba, and Praia do Forte and called the Southern group (Figure 2).

Estimates of the fixation index (*f*), with an overall mean of 0.043 and values of the apparent outcrossing rates (t_a_) around the overall mean of 0.918, in addition to results of the test of goodness of fit to Hardy-Weinberg equilibrium, demonstrate that the majority of the populations studied reproduce predominantly by panmixia.

The present study permitted the conclusion that microsatellite markers are effective in estimating genetic variation levels within and between the populations of Brazilian Tall coconut trees. Brazilian populations exhibited high genetic divergence detected by the employed markers. Diversity among the investigated populations is spatially structured, with a greater similarity among geographically close populations. The studied populations of Brazilian Tall coconut are preferentially allogamous, with a mean apparent outcrossing rate of 92%. Taken together, our results will provide important tools for *ex situ* germplasm conservation, selection, and support of breeding programs in Brazil.

## Figures and Tables

**Table 1:** Brazilian populations of Tall coconut (*Cocos nucifera* L.) investigated in the present study of genetic diversity by microsatellite markers (SSR). Geographic coordinates, plant height and the number of plants are also shown.

Population	No of plants	Location (State)	Latitude (S)	Longitude (W)	Height (m)
1. Santo Inácio (BRAsi)	17	Maranhão	02°34'54”	42°45'15”	2
2. Luís Correia (BRAlc)	17	Piauí	02°57'59”	41°35'45”	7
3. Baía Formosa (BRAbf)	30	Rio Grande do Norte	06°23'14”	35°03'31”	9
4. Georgino Avelino (BRAga)	21	Rio Grande do Norte	06°10'57”	35°06'02”	2
5. São José do Mipibu (BRAsjm)	18	Rio Grande do Norte	06°06'43”	35°14'36”	8
6. Santa Rita (BRAsr)	21	Pernambuco	07°10'54”	34°53'01”	11
7. Merepe (BRAme)	20	Pernambuco	08°28'38”	34°59'52”	2
8. Japoatã (BRAjp)	18	Sergipe	10°27'08”	36°42'42”	232
9. Pacatuba (BRApc)	19	Sergipe	10°30'29”	36°35'37”	6
10. Praia do Forte (BRApf)	14	Bahia	12°34'09”	37°59'48”	2

**Table 2:** Number of alleles per locus, variation in allele length (bp), gene diversity (H_e_), and observed heterozygosity (H_o_) estimated for 10 populations of Brazilian Tall coconut trees, using 13 SRR loci.

SSR Locus	Allele number	Allele length (bp)	H_e_	H_o_
CnCir A3	3	228-240	0.034	0.036
CnCir A9	4	089-103	0.513	0.484
CnCir B6	5	196-208	0.613	0.531
CnCir B12	9	157-181	0.515	0.464
CnCir C7	5	157-167	0.495	0.470
CnCir C12	6	163-183	0.378	0.355
CnCir E2	13	115-165	0.711	0.671
CnCir E10	4	232-246	0.418	0.443
CnCir E12	2	164-174	0.300	0.302
CnCir F2	3	193-205	0.510	0.536
CnCir G11	7	188-210	0.638	0.640
CnCir H4'	3	218-230	0.330	0.311
CnCir H7	4	133-141	0.512	0.518
Mean	5.23		0.459	0.443
Total	68			

**Table 3:** Estimates of genetic parameters for 10 populations of Brazilian Tall coconut trees. Confidence interval (CI) at 95% probability.

	f	θ_P_	F	R_ST_
Estimate	0.043	0.160	0.196	0.086
Lower limit (CI 95%)	0.013	0.122	0.151	0.052
Upper limit (CI 95%)	0.073	0.199	0.234	0.130

**Table 4:** Matrix of Nei genetic (diagonal top half) and geographic distances in km (diagonal bottom half) of pairwise comparisons of 10 populations of Brazilian Tall coconut.

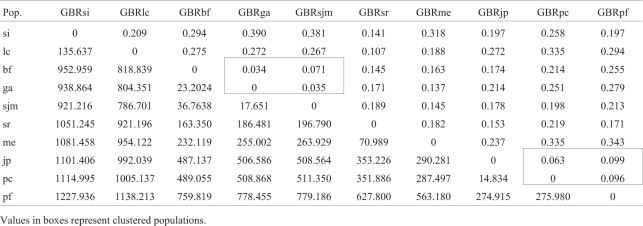

Values in boxes represent clustered populations.

**Table 5:** Estimates of the intrapopulation fixation index (*f*) with corresponding confidence interval (95%) and apparent outcrossing rate (t_a_) for the 10 investigated populations of Brazilian Tall coconut.

Population	F	Lower limit	Upper limit	t_a_
GBRsi	-0.06577	-0.29560	0.07134	-
GBRlc	0.01819	-0.16604	0.12592	0.965
GBRbf	0.09898	-0.02575	0.19301	0.820
GBRga	0.12301	-0.04205	0.21392	0.781
GBRsjm	0.13367	-0.04707	0.25312	0.764
GBRsr	-0.05849	-0.21674	0.02021	-
GBRme	0.10948	-0.09987	0.25301	0.803
GBRjp	0.05944	-0.11711	0.18039	0.888
GBRpc	-0.09966*	-0.25122	-0.03124	-
GBRpf	0.08470	-0.08320	0.16329	0.843
Mean	0.0426	0.0108	0.0731	0.918

*Statistically significant at a 5% probability.
